# The complete chloroplast genome of *Ardisia mamillata* (Myrsinaceae)

**DOI:** 10.1080/23802359.2019.1673680

**Published:** 2019-10-09

**Authors:** Na Hou, Mao Li, Jingmin Shen, Zhiping Chen, Yang Luo, Lunxiu Deng

**Affiliations:** Guizhou Academy of Forestry, Guiyang, Guizhou Province, People’s Republic of China

**Keywords:** *Ardisia mamillata*, high-throughput sequencing, chloroplast genome

## Abstract

The chloroplast genome sequence of *Ardisia mamillata* has been characterized based on the High-throughput sequencing technology. The complete cp genome of *A. mamillata* is 138,323 bp in length, containing LSC region of 86,325 bp, SSC region of 18,434 bp, and two IR regions of 25,999 bp. The overall GC content f *A. mamillata* cp genome is 37.1%. The annotated complete cp genome contains 113 genes, including 79 protein-coding genes, 8 rRNA genes, and 30 tRNA genes. Further, the phylogenetic analysis suggested that the *A. mamillata* and *A. polysticta* are phylogenetically related to each other.

*Ardisia mamillata* Hance (Myrsinaceae) is a kind of rare ornamental shrub with high medicinal value and widely distributes in Southern China. The roots of *A. mamillata* have been traditionally used to treat respiratory tract infections and menstrual disorders (Huang et al. 2000). However, the phylogenetic position of *A. mamillata* and the genus *Ardisia* is still unresolved. In this project, we first reported the complete chloroplast (cp) genome of *A. mamillata* based on the Illumina pair-end sequencing.

The *A. mamillata* was collected at country Rongjiang, City Guiyang, province Guizhou, China (E108°13′38.25″, N25°43′33″) and the voucher specimen was stored at Guizhou Academy of Forestry (HSH-2017-6-30). The total genomic DNA was extracted from a single fresh leaf using DNeasy Plant Pro Kit (Aidlab, Beijing, China) following the manufacturer's guidelines. 4 μg DNA was used to sequence based on the Illumina pair-end technology. Totally, 122.9 M of 125-bp clean paired reads were obtained after raw data quality trimming. The cp genome of *A. mamillata* was assembled using the program MITObim v1.9 (Hahn et al. 2013) with complete chloroplast genome of Ardisia polysticta) (GenBank accession NO. KC465962) (Ku et al. 2013) as the reference. The assembled *A. mamillata* cp genome was annotated using the online tool (DOGMA) (Wyman et al. 2004). The new annotated complete cp genome was submitted to GenBank and get the accession number MN136062. The complete cp genome of *A. mamillata* is 156,757 bp in length, containing an LSC region of 86,325 bp, an SSC region of 18,434 bp, and two IR regions of 25,999 bp. The overall GC content f *A. mamillata* cp genome is 37.1%. The annotated complete cp genome contains 113 genes, including 79 protein-coding genes, 8 rRNA genes, and 30 tRNA genes. Among all of these genes, 18 genes contains introns, including 12 protein-coding genes (*atpF*, *clpP*, *ndhA*, *ndhB*, *petB*, *petD*, *rpl2*, *rpl16*, *rpoC1*, *rps12*, *rps16*, and *ycf3*) and 6 tRNA (*trnA-UGC*, *trnG-UCC*, *trnI-GAU*, *trnK-UUU*, *trnL-UAA*, and *trnV-UAC*).

To further investigate the phylogenetic position of *A. mamillata*, a Neighbor-Joining tree was carried out based on with 10 protein-coding genes extracted from the complete chloroplast genome sequences of 18 species within the family Primulaceae using MEGA X (Kumar et al. 2018) with 1000 bootstrap replicates. Three *Diospyros* species within the family Ebenaceae were included as outgroup taxa. Our results showed that *A. mamillata* and *A. polysticta* are phylogenetically related to each other (Figure 1).

**Figure 1.**
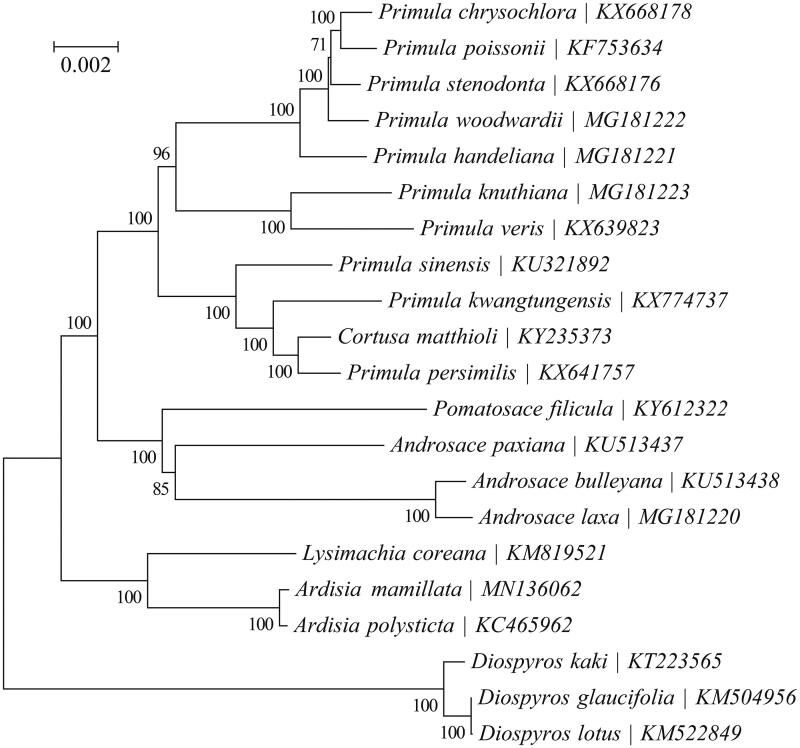
Phylogeny of 18 species within the family Primulaceae based on the neighbor-joining (NJ) analysis of the concatenated chloroplast protein-coding sequences. The support values are based on 1000 bootstrap replicates and are placed next to the branches. Three *Diospyros* species within the family Ebenaceae were included as outgroup taxa.
